# Strong Depth-Related Zonation of Megabenthos on a Rocky Continental Margin (∼700–4000 m) off Southern Tasmania, Australia

**DOI:** 10.1371/journal.pone.0085872

**Published:** 2014-01-22

**Authors:** Ronald Thresher, Franziska Althaus, Jess Adkins, Karen Gowlett-Holmes, Phil Alderslade, Jo Dowdney, Walter Cho, Alex Gagnon, David Staples, Felicity McEnnulty, Alan Williams

**Affiliations:** 1 CSIRO Wealth from Oceans Flagship, Hobart, Tasmania, Australia; 2 California Institute of Technology, Pasadena, California, United States of America; 3 Woods Hole Oceanographic Institution, Woods Hole, Massachusetts, United States of America; 4 Museum of Victoria, Melbourne, Victoria, Australia; University of Glasgow, United Kingdom

## Abstract

Assemblages of megabenthos are structured in seven depth-related zones between ∼700 and 4000 m on the rocky and topographically complex continental margin south of Tasmania, southeastern Australia. These patterns emerge from analysis of imagery and specimen collections taken from a suite of surveys using photographic and *in situ* sampling by epibenthic sleds, towed video cameras, an autonomous underwater vehicle and a remotely operated vehicle (ROV). Seamount peaks in shallow zones had relatively low biomass and low diversity assemblages, which may be in part natural and in part due to effects of bottom trawl fishing. Species richness was highest at intermediate depths (1000–1300 m) as a result of an extensive coral reef community based on the bioherm-forming scleractinian *Solenosmilia variabilis*. However, megabenthos abundance peaked in a deeper, low diversity assemblage at 2000–2500 m. The *S. variabilis* reef and the deep biomass zone were separated by an extensive dead, sub-fossil *S. variabilis* reef and a relatively low biomass stratum on volcanic rock roughly coincident with the oxygen minimum layer. Below 2400 m, megabenthos was increasingly sparse, though punctuated by occasional small pockets of relatively high diversity and biomass. Nonetheless, megabenthic organisms were observed in the vast majority of photographs on all seabed habitats and to the maximum depths observed - a sandy plain below 3950 m. Taxonomic studies in progress suggest that the observed depth zonation is based in part on changing species mixes with depth, but also an underlying commonality to much of the seamount and rocky substrate biota across all depths. Although the mechanisms supporting the extraordinarily high biomass in 2000–2500 m depths remains obscure, plausible explanations include equatorwards lateral transport of polar production and/or a response to depth-stratified oxygen availability.

## Introduction

Depth-related environmental factors structure marine benthic communities in the deep ocean, reflecting a well-documented general zonation along a shore to deep-ocean axis [1]–[5]. Up to seven biotic zones have been described along this axis, based on changes in species composition, abundance and trophodynamics [3], [6]. Transitions between zones can be relatively abrupt, and often coincide with geological features. The mechanisms that underlie this depth zonation are diverse and likely to differ between locations and between taxa [3], [5]. They can also be difficult to separate due to limited scope for experimental manipulation [7]. However, four generalities emerge. First, biomass and density tend to decrease with depth, with the noteworthy exception of hydrothermal vent communities [8]. This is generally attributed to declining availability of surface-derived production with increasing depth [3], [4]. Second, single species or small groups of species can numerically dominate, and hence to a large extent, define particular zones [6], [9]. Third, species richness tends to peak at intermediate depths [2], [3], [9] but fourth, declines overall with increasing depth [7], [10], [11].

The extent to which these generalities apply to seamount (seamounts, knolls, hills) communities is poorly known. Seamounts have been suggested to be centers of benthic biomass, diversity and possibly endemism in the deep ocean [12], and hence could provide opportunities to test general hypotheses about bathymetric structuring over small spatial scales and, often, relatively homogeneous substrates [13]. However, recent reviews of seamount benthic communities barely refer to bathymetric structuring [12], [14]. This absence may reflect studies that until recently have been limited by sampling constraints to relatively shallow depths, typically less than 1500 m. Early work on seamount biota appeared to confirm expectations derived from general deep-sea studies that the abundance and diversity of benthic organisms decreased with increasing depth [15], [16]. However, several recent studies span a wider depth range and report not only seamount assemblages that are zoned by depth, but also weak or non-linear relationships between depth and benthos abundance and diversity [13], [17], [18]. Such zonation, if widespread, needs to be factored into global analyses of seamount diversity and an understanding of the mechanisms that underpin it [13]. More broadly, the scarcity of information on deep seamount biota is of concern in the face of increasing anthropogenic impacts on deep-sea ecosystems [5], [19].

**Figure 1:**
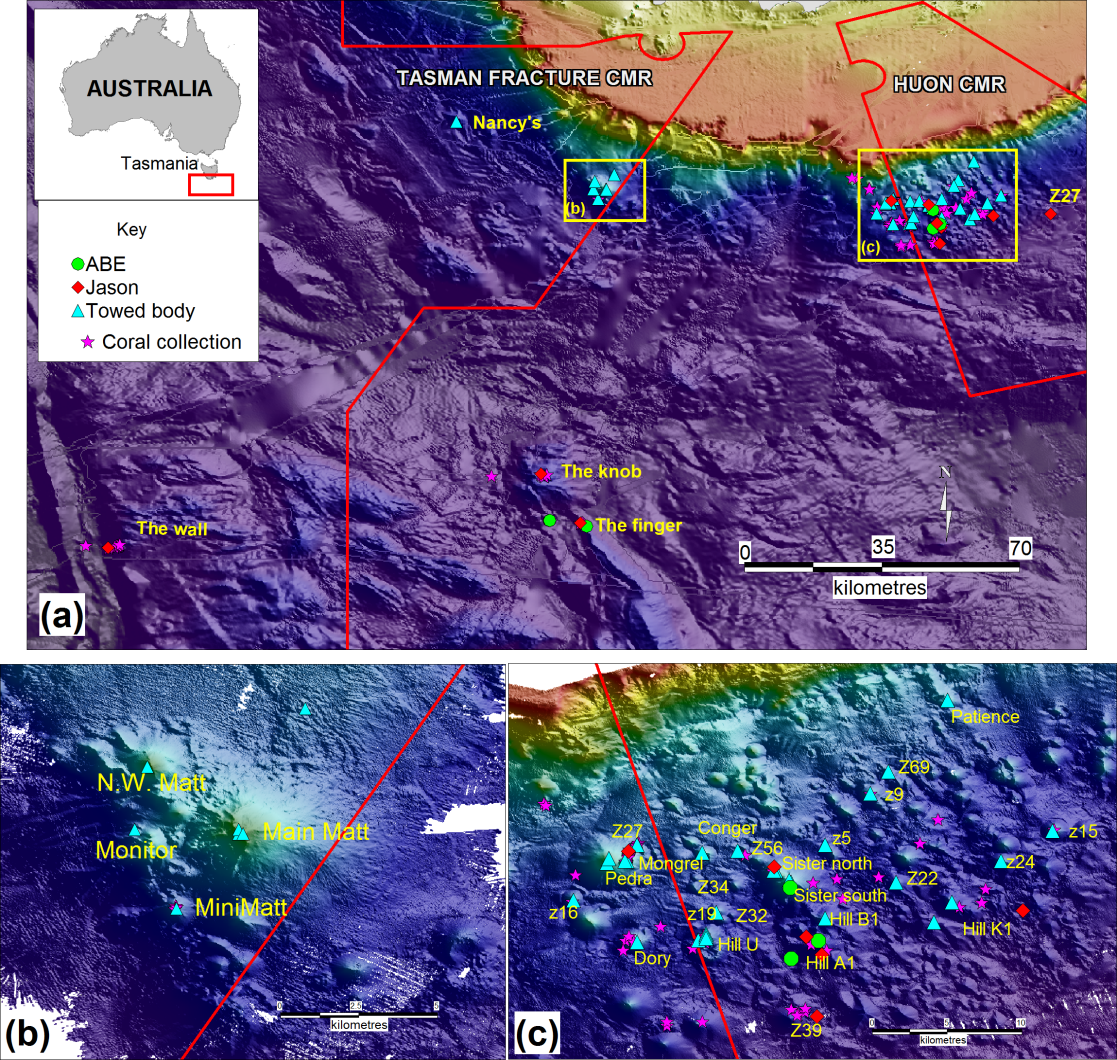
Dive, trawl and towed body survey locations off south and eastern Tasmania, overlaid on a rendered swath bathymetry map (yellow-100 m to dark purple- 4000 m; Geoscience Australia 2009 & CSIRO survey data). CMR = Commonwealth Marine Reserve.

In this paper, we describe and semi-quantitatively analyze seamount megabenthos assemblages on seamounts and associated rocky substrate along the continental margin of Tasmania, Australia to a depth of over 4 km (Fig. 1). Koslow et al. [16], Althaus et al [20] and Rowden et al. [21] describe the morphology, geology and shallow benthic ecology of these mostly small seamounts. Our descriptions and analyses are integrations of three sampling series that overlap in depth ranges: epibenthic sled sampling and video and still images taken using a towed camera that covers a depth range from the shallowest seamount peaks (646 m) to 1817 m; an autonomous underwater vehicle (AUV) survey spanning 820 to 2950 m; and a set of remotely operated vehicle (ROV) dives than span 941 to 4011 m. We acknowledge that integrating these data sets has the potential to introduce significant sampling artifacts into the analyses, but also suggest that in combination they can indicate robust trends in depth-related changes in biodiversity on seamount and associated hard-ground features, that are globally poorly sampled and that span more than a 3 km depth range. The combined data set includes over 30,000 photographs, 500 hours of video, and 230 hours of AUV and ROV bottom time, plus extensive benthic sampling for taxonomic and paleo-climatic analyses. From these data, we (1) describe depth-related patterns in benthic mega-faunal assemblages, (2) compare our observations with the information available for deep continental margin assemblages elsewhere, and (3) discuss the production sources likely to be supporting these assemblages.

## Methods

### Study area and survey data

The data presented here stem from four surveys off southern Tasmania (Fig. 1). Surveys SS2006/11 and SS2007/02 were done using the Australian National Facility RV *Southern Surveyor* and employed multi-beam sonar mapping, towed camera imaging and epibenthic sled sampling to assess seamount biota to a depth of 1817 m. Survey SS2008/01 also employed the RV *Southern Surveyor*, but deployed the Woods Hole Oceanographic Institute AUV *ABE (Autonomous Benthic Explorer)* on seamount features to 2950 m. The fourth survey was done using the US RV *Thomas G. Thompson* (TN 228) to deploy the Woods Hole Oceanographic Institute ROV *Jason2* (referred to hereafter as *Jason*) on seamount and associated hard ground features to 4011 m. Details of the towed camera surveys, *ABE* and *Jason* dives are given in Table 1. In aggregate, the *ABE* and *Jason* dives span 6 seamounts in and near the Australian Huon Commonwealth Marine Reserve (CMR), two abrupt rocky features (the “Knob” and the “Finger”) just east of the Tasman Fracture CMR, and a rocky slope (the “Wall”), from 2386 to 4011 m, along the western edge of the Tasman Fracture CMR. Field work in the marine reserves was authorized by the Australian Department of Environment, Water, Heritage and the Arts, permit number AU-COM2008044. *Jason* dives outside of this area that are referred to in the text were made at the Cascade Plateau, a large, isolated seamount in the mid-Tasman Sea (dive 390, at 43° 48.2′S; 150° 19.2′E) and on a small unnamed seamount off the northeast coast of Tasmania (dive 389, at 41° 14.3′S; 148° 49.1′E). Epibenthic sled and towed camera surveys encompassed seamounts in both the Huon and Tasman Fracture CMRs. Field data, results of analyses and specimens not dispersed to taxonomic experts are archived at the CSIRO Marine Laboratory.

**Table 1:** Summary of towed camera surveys and *ABE* and *Jason* dive programs.

Vehicle	Dive #	Date (local)	Location, CMR	Latitude; Longitude	Depth (m)	Bottom Time (h:mm)	No. still images scored	Depth range of scored images (m)
Towed camera	39	5/11/06	B1, Huon	44° 18.4′S; 147° 16.8′E	1070–1600	0:31	60	1139–1405
Towed camera	35	5/11/06	Conger, Huon	44° 15.1′S; 147° 10.6′E	990–1287	0:23		
Towed camera	24	3/11/06	Dory, west of Huon	44° 19.6′S; 147° 7.3′E	1122–1424	0:24	111	1091–1469
Towed camera	30	5/11/06	K1, Huon	44° 18.6′S; 147° 22.3′E	1235–1775	0:26	98	1274–1817
Towed camera	31	5/11/06	K1, Huon	44° 17.6′S; 147° 23.2′E	1235–1600	0:20	13	1337–1391
Towed camera	40	5/11/06	K1, Huon	44° 17.6′S; 147° 23.2′E	1270–1650	0:20	25	1248–1390
Towed camera	44	6/11/06	Z27, west of Huon	44° 14.7′S; 147° 7.3′E	1065–1240	0:16		
Towed camera	34	5/11/06	Little Sister (Z56), Huon	44° 15′S; 147° 12.4′E	1080–1297	0:21		
Towed camera	64	8/11/06	Main Matt, TF	44° 14.7′S; 146° 9.9′E	680–1138	0:28	6*	
Towed camera	66	9/11/06	Main Matt, TF	44° 12.9′S; 146° 11.4′E	646–646	0:02		
Towed camera	67	9/11/06	Main Matt, TF	44° 12.8′S; 146° 8.9′E	660–1060	0:31		
Towed camera	70	9/11/06	Main Matt, TF	44° 12.8′S; 146° 11.4′E	690–1200	0:46		
Towed camera	71	9/11/06	Main Matt, TF	44° 9.9′S; 146° 13′E	700–1170	1:31		
Towed camera	65	8/11/06	Mini Matt, TF	44° 12.9′S; 146° 11.5′E	1135–1430	0:16	44	1178–1442
Towed camera	22	2/11/06	Mongrel, west of Huon	44° 15.5′S; 147° 6.8′E	730–1225	0:59	207*	
Towed camera	43	6/11/06	Mongrel, west of Huon	44° 15.5′S; 147° 6.7′E	779–1130	0:34	84*	
Towed camera	69	9/11/06	Monitor, TF	44° 12.9′S; 146° 11.5′E	938–1400	0:27	32*	
Towed camera	74	9/11/06	Nancy's, TF	43° 59.6′S; 145° 42.2′E	1430–1805	0:51		
Towed camera	25	4/11/06	North Sister, Huon	44° 16′S; 147° 14.3′E	822–1340	0:31	81*	
Towed camera	33	5/11/06	North Sister, Huon	44° 16′S; 147° 14.2′E	912–1270	0:28	73*	
Towed camera	63	8/11/06	NW Matt, TF	44° 11.3′S; 146° 9.2′E	808–1235	0:40	41*	
Towed camera	54	7/04/07	Patience, Huon	44° 7.4′S; 147° 23′E	900–1250	0:23	124*	
Towed camera	20	2/11/06	Pedra, west of Huon	44° 15.6′S; 147° 5.8′E	840–1400	1:08	180*	
Towed camera	36	5/11/06	Pedra, west of Huon	44° 15.4′S; 147° 5.9′E	770–1335	0:57	108*	
Towed camera	19	2/11/06	South Sister, Huon	44° 16.6′S; 147° 15.1′E	834–1402	0:48		
Towed camera	41	5/11/06	South Sister, Huon	44° 16.5′S; 147° 15′E	923–1340	0:39	161*	
Towed camera	21	2/11/06	U, Huon	44° 19.5′S; 147° 10.4′E	1327–1470	0:24	12	1352–1401
Towed camera	23	3/11/06	U, Huon	44° 19.2′S; 147° 10.8′E	1100–1444	0:24	69	1114–1413
Towed camera	27	4/11/06	U, Huon	44° 19.3′S; 147° 10.8′E	1130–1335	0:21	96	1099–1323
Towed camera	38	5/11/06	U, Huon	44° 19.4′S; 147° 10.8′E	1130–1470	0:50	87	1114–1399
Towed camera	28	4/11/06	Z15. Huon	44° 14′S; 147° 28.3′E	1090–1600	0:26		
Towed camera	37	5/11/06	Z16, west of Huon	44° 17.5′S; 147° 4.1′E	1020–1434	0:20	55	1057–1404
Towed camera	26	4/11/06	Z19. Huon	44° 18.1′S; 147° 11.3′E	1070–1400	0:15		
Towed camera	32	5/11/06	Z22. Huon	44° 16.6′S; 147° 20.4′E	1179–1480	0:14		
Towed camera	29	4/11/06	Z24. Huon	44° 15.5′S; 147° 25.7′E	1166–1600	0:20		
Towed camera	42	5/11/06	Z5, Huon	44° 14.7′S; 147° 16.8′E	1291–1406	0:07	22	1176–1250
Towed camera	55	7/04/07	Z69. Huon	44° 11′S; 147° 20.0′E	1150–1280	0:11		
Towed camera	56	7/04/07	Z9. Huon	44° 12.1′S; 147° 19.1′E	1010–1300	0:16		
*ABE*	211	15/01/08	South Sister, Huon	44° 19.54′S; 147° 16.5′E	1414	1:30		
*ABE*	212	17/01/08	South Sister, Huon	44° 16.86′S; 147°15.0′E	820–1050	18:09		
*ABE*	213	18/01/08	A1, Huon	44° 20.44′S; 146° 15.1′E	1600–1900	20:03		
*ABE*	214	22/01/08	TFZ	45° 17.28′S; 146° 0.3′E	2630–2950	8:25		
*ABE*	215	23/01/08	The Finger, TF	45° 17.28′S; 146° 0.4′E	2600–2700	2:20		
*ABE*	216	25/01/08	The Finger, TF	45° 18.46′S; 146° 7.6′E	2080–2500	13:57		
*Jason*	382	17/12/08	A1, Huon	44° 20.24′S; 146° 53.1′E	1305–1689	7:20		
*Jason*	383	18/12/08	A1, Huon	44° 19.35′S; 146° 15.9′E	1291–1575	12:30		
*Jason*	384	19/12/08	North Sister, Huon	44° 15.8′S; 147° 14.2′E	941–958	10:47		
*Jason*	385	21/12/08	Z27, Huon	44° 17.6′S; 147° 38.0′E	1061–1240	10:54		
*Jason*	386	24/12/08	Mongrel, Huon	44° 15.03′S; 147° 6.9′E	729–1109	17:40		
*Jason*	387	26/12/08	Z39, Huon	44° 23.35′S; 147° 16.4′E	1439–2051	48:05	74	1652–1997
*Jason*	391	9/01/09	The Wall, TF	45° 22.57′S; 144° 34.4′E	2386–4011	16:14	267	2233–4011
*Jason*	392	11/01/09	The Finger, TF	45° 17.75′S; 146° 06.3′E	2213–2898	14:50	111	2222–2898
*Jason*	393	12/01/09	The Knob, TF	45° 08.21′S; 145° 58.7′E	1410–1803	14:53		
*Jason*	395	14/01/09	K1, Huon	44° 18.02′S; 147° 26.8′E	1230–2194	23:58	69	1503–2194

Towed body surveys marked with a * were on commercially fished seamounts and not used in the semi-quantitative analyses. Huon  =  Huon CMR; TF  =  Tasman Fracture CMR.

### Imagery collection

Images from the CSIRO towed camera system were captured using fixed angle/zoom stereo video cameras and a high-resolution digital still camera with an oblique fixed field of view. The camera platform was towed 2–4 m above the bottom at ∼1 knot (0.5 m/sec), in a down-slope direction, resulting in more or less straight-line transects. High-resolution digital still images were taken at ∼10 second intervals and the stereo configuration of the video cameras was used to draw polygons of known absolute areas onto each still image [22]. All images were used unless they were poor quality, because the camera was too far off bottom or because the image was under/over exposed or out of focus. The 10-second interval between images resulted in no overlapping of images. The measured field of view averaged 5.4 m^2^.

**Figure 2:**
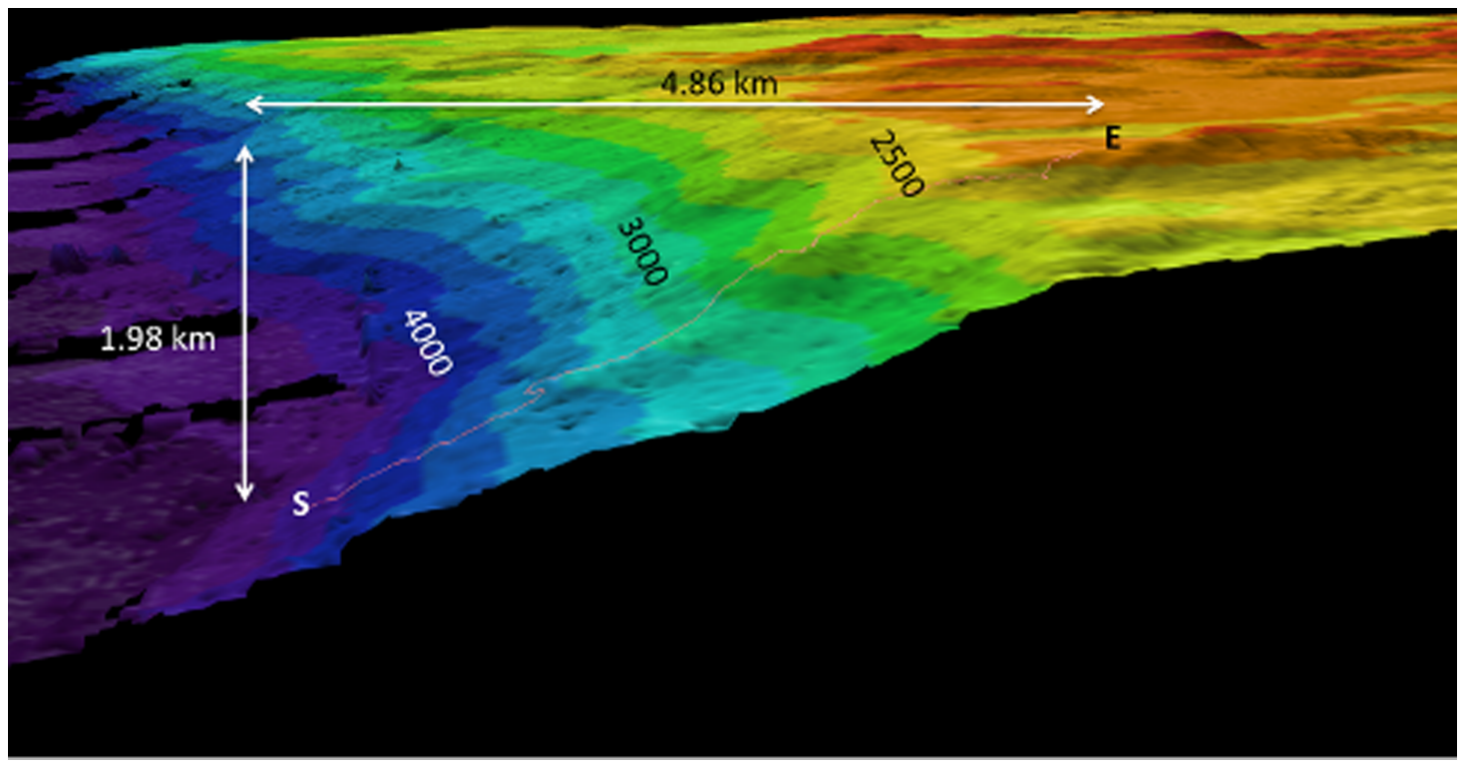
Track of *Jason* dive #391 (irregular red line) on a swath map of “The Wall” in the Tasman Fracture Zone, the latter produced using a hull-mounted Simrad EM 302 multibeam echo sounder. The dive started (S) at 4011 m and ended (E) at 2386 m, and spanned 16.1 hours. Color bands mark 200 m depth intervals (dark blue = 3800 – 4000 m; light green = 2800–3000 m; orange 2200 – 2400 m).

Images for the deeper transects were taken using two *Jason* cameras. All *Jason* transects were done in an up-slope direction, in a generally straight-line direction (e.g., Fig. 2), but the vehicle was stopped frequently for sample collection and to investigate particular features, which often included horizontal movements along the substratum. The “light bar” camera (a composite 1-chip 6.5 kM wide angle Pegasus video camera) took frame grabs automatically at 2 minute intervals during the dives, as well as at irregular intervals when triggered manually; on average, an image was taken every 56 seconds. Data for community analyses were taken from a sub-set of the light-bar images for each dive by *a priori* and randomly subsampling the locations of individual images plotted in a GIS to approximate a straight-line up-slope transect. This eliminated over-sampling bias stemming from targeted photography of particular seabed features and while *Jason* travelled along rather than up bathymetric contours, and was consistent with the towed camera sampling. *Jason* also took close-up images using a manually triggered 1-chip, color high definition television camera custom built by the Woods Hole Advanced Imaging and Visualization Laboratory. This camera was panned, tilted and zoomed as required, while the light bar camera covered a fixed angle relative to the vehicle and hence a relatively constant oblique field of view. Only images taken using the light bar camera were used in the semi-quantitative analysis of community structure. The average area of the seafloor in the field of view was estimated to be similar to that of towed camera system. Details about *Jason* and its instrumentation are available at www.whoi.edu/nsfVehicles/Jason/. Mission details, data and a large number of images taken during the dive program using the *Jason* cameras are available at http://4dgeo.whoi.edu/jason, under 2008, tn228 and Virtual Van.

*ABE* was programmed to navigate 3 m above the substratum, which produced a planar image of about 16 m^2^. The *ABE* and its instrumentation are described by German et al. [23] and www.whoi.edu/page.do?pid=8458.

### Water column parameters

These were determined by hydrocasts, complemented by near bottom measurements made using CTDs mounted on the *ABE* and *Jason*. Profiles of temperature, salinity and carbonate chemistry derived from hydrocasts closely matched values taken just off the substratum using the vehicles [24], [25]. For a qualitative comparison between seamount assemblages and water column structure, we graphed the data from the deepest hydrocast (3501 m), taken in the Tasman Fracture CMR at 44° 50′S, 145° 45′E on 10 Jan 2009, against the identified faunal depth structure.

### Analyses of bathymetric structure in megabenthic communities

#### Image selection for community structure analyses

**Figure 3:**
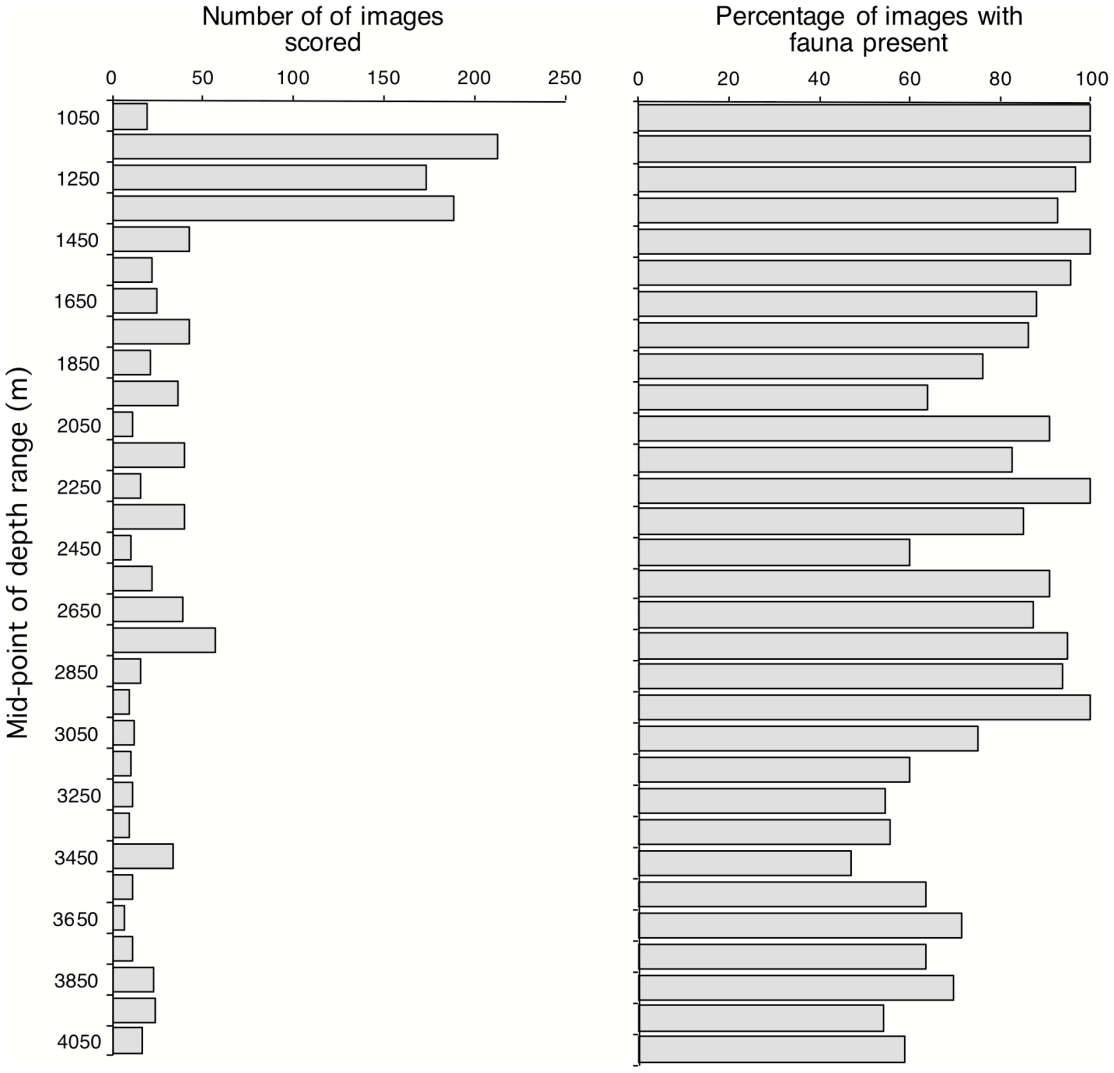
Distribution by depth (100 m intervals) of (a) the number of images scored and (b) the percentage of images in which benthic megafauna was observed.

Community composition was assessed semi-quantitatively using 1213 images from 16 towed camera and Jason transects scored for substratum type and biota. Images were taken on 7 seamounts (towed body and Jason - 761 images from 13 transects, spanning 1057–2194 m) and three deep rocky features - the Finger, Knob and Wall (Jason - 452 images from 3 transects, spanning 1652–4011 m) (Table 1). For Jason, we used only non-overlapping images, eliminated duplicates (i.e., where Jason was stationary), and excluded images that were too dark, clearly taken well off bottom or were out of focus. Data were taken from an image sub-set for each dive by a priori and randomly subsampling the locations of individual images plotted in a GIS to approximate a straight-line up-slope transect. This eliminated over-sampling bias stemming from targeted photography of particular seabed features and while Jason travelled along rather than up bathymetric contours, and was consistent with the towed camera sampling. The depth distribution of all other scored images is shown in Fig. 3.

An additional 1097 images taken on seamount peaks (<∼900 m) using the towed camera were scored as part of the overall description of the seamount benthos (Table 1), but not used in the semi-quantitative analysis. The shallow seamount peaks are likely, or known to be impacted by commercial fishing [20] and hence their benthic assemblages reflect the impact of factors other than a natural zonation of communities by depth. Estimates of megabenthos density were taken from images produced by *ABE*'s downward looking, 1024×1024 pixel, 12-bit digital color still camera. However, they were not included in the semi-quantitative analyses, because the sampling approach and viewing angles were not comparable to the other methods.

#### Megabenthos and habitat identification

Each image was scored for dominant substrata (sediment, rock/cobble or biogenic), geomorphology and structural habitat type (see [20] for criteria and methodology), as well as estimated percent cover of three biogenic substrata: (1) intact three-dimensional coral matrix composed of live or dead Solenosmilia variabilis; (2) coral rubble; and (3) barnacle plates. We also recorded the number of individuals in each image of 67 different invertebrate taxa that we felt we could reliably identify in the still images (see results for list of taxa) following the photo taxonomy used in [20] and expanding on it to include additional taxa identified in images taken from the Jason. The counts were semi-quantitative, being of all individuals of identifiable taxa for the entire field of view where possible, but only counting a sub-set of the area and scaling the count up where we encountered more than about 100 individuals of the same apparent taxon in a single image. Because camera angle could change somewhat between images with heave of the camera platform, particularly for Jason, we recorded a qualitative assessment of the area of the field of view and used this to normalize each count to a roughly comparable sample area. Identifying biota in the Jason images was facilitated by examining images taken with the high definition camera where they overlapped those taken with the light bar camera. Identifications were also checked against samples collected using the epibenthic sled at depths less than about 1800 m, and targeted collections made using grab, scoop and suction samplers deployed using Jason. Samples were sorted on board into identifiable taxonomic units, photographed and then preserved, usually in 70% ethanol. Preserved specimens were distributed to taxonomic experts and museums, and identified to the level currently possible for each taxon. The most highly resolved groups to date are the octocorals (by P. Alderslade, CSIRO), decapods (by G. Poore, Museum of Victoria) and ophiuroids (by T. O'Hara, Museum of Victoria). For these groups, epibenthic sled and Jason-derived samples were crosschecked for consistency of identification between sampling methods and surveys. Collection depths are precisely known for the Jason samples, but were the mid-point of the depth range of the epibenthic sled tow. Most sled tows spanned a range of <500 m.

#### Statistical analysis

The images collected by the towed camera system and by the Jason light bar camera were considered comparable enough in regard to the angle and field of view sampled to warrant combining the count per image data from the two methods for semi-quantitative analyses. For data summaries and statistical analyses the counts per image data was binned into 31 100 m depth strata from 1000–1100 m to 400–4100 m (ranges are lower limit inclusive, i.e. < = 1000 to <1100 m, etc.).

Four community metrics are presented for 100 m depth strata: (1) the percentage of images with evident megafauna; (2) the number of individuals per image (average and range); (3) photo-taxon richness, per image (average and range) and cumulative per depth stratum; and (4) the Shannon-Wiener diversity (H′). Correlations (Spearman Rank - r_s_) against depth and other parameters were calculated using Statview. We also present and discuss summary statistics per depth stratum.

Multivariate statistics were used to identify bathymetric patterns in the community structure. Group averaged cluster analysis and non-mentric multidimensional scaling (nMDS) were done in Primer v6 (www.primer-e.com/primer.htm) on the average number of individuals per image of all scored taxa in each 100 m depth stratum. Data were square root transformed prior to analysis to reduce the influence of the dominant taxa, and the Bray-Curtis similarity index was used. The Similarity Profile Test (SIMPROF) routine in Primer informed the decision regarding the final number of clusters; cluster splits that are interchangeable at p>0.05 were considered non-significant and are shown visually as red lines in the cluster tree. Species accumulation curves (averaged over 999 random permutations of images in each group) were used to compare the diversity of the significantly different cluster groups.

The bathymetric zonation of the megabenthos communities identified in the statistical analyses were discussed in light of qualitative observations made from all images and video footage collected using *ABE*, *Jason* and the towed camera.

## Results

### Depth-related trends in habitats and megabenthos

**Figure 4:**
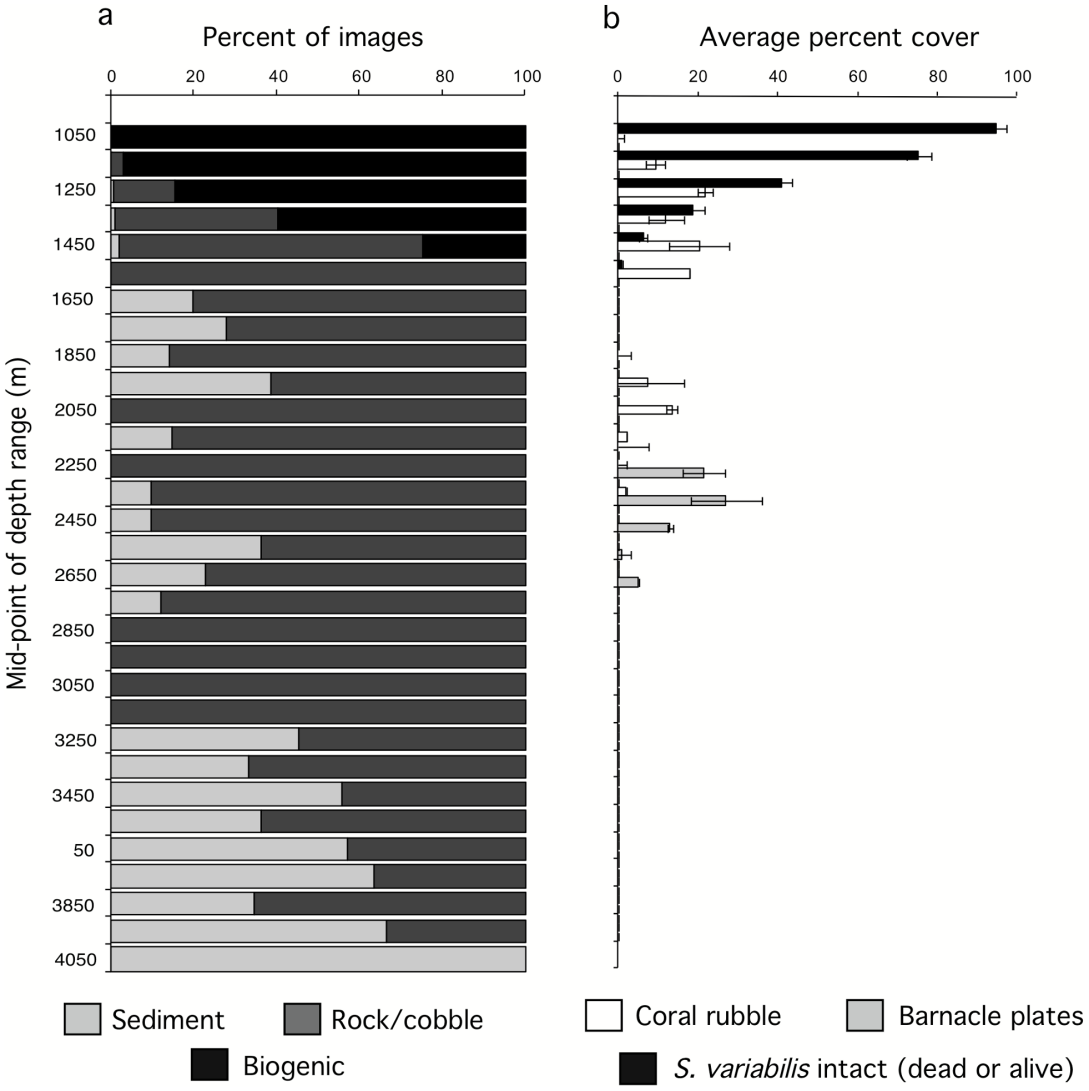
(a) Distribution of the dominant substrate and dominant geomorphology types over the depth categories (expressed as % of all scored images). (b) Distribution over the depth categories of the average of the percentage cover recorded for intact *Solenosmilia* matrix (dead & alive), coral rubble and barnacle plates. Horizontal lines indicate standard error of the averages in each depth category.

Consistent depth-related trends were apparent in data summary statistics and all four univariate metrics of megabenthos assemblage composition. Trends held for the relatively sparse data taken below 3000 m (average of 15.3 images scored per 100 m). Four trends can be summarized as follows:

First, megabenthos was observed in most images across the depth range surveyed (∼600 to 4000 m). Over the 1213 images scored, 88% showed at least one megabenthic organism. Across the 31 100-m depth bins analyzed, means ranged from a maximum of 100% (at 1000–1300 m, 1400–1500 m, 2200–2300 m and 2900–3000 m) to a minimum of 47.0% at 3400–3500 m (Fig. 3b). The proportion of images with conspicuous megabenthos decreased with depth (r_s_ = 0.72, p<0.001), but even at 4000+ m, 59% of 17 images scored still contained apparently live megabenthos. The overall decrease with depth correlates negatively with the proportion of images in each depth stratum in which sediment was the dominant substratum (r_s_ = 0.67, p<0001), confirming field observations that most of the observed megafauna was on hard substrata, either rock or of biogenic origin. The latter dominated the images only near and immediately below the peak of the *Solenosmilia* reef zone, between 1000–1300 m (Fig. 4), but it was also conspicuous in two other depth zones, a patchy coverage of dead coral and coral rubble (presumably derived from *Solenosmilia*) between roughly 1250–1600 m, and a zone between 2300–2700 m with extensive windrows of barnacle plates (Fig. 4b).

**Figure 5:**
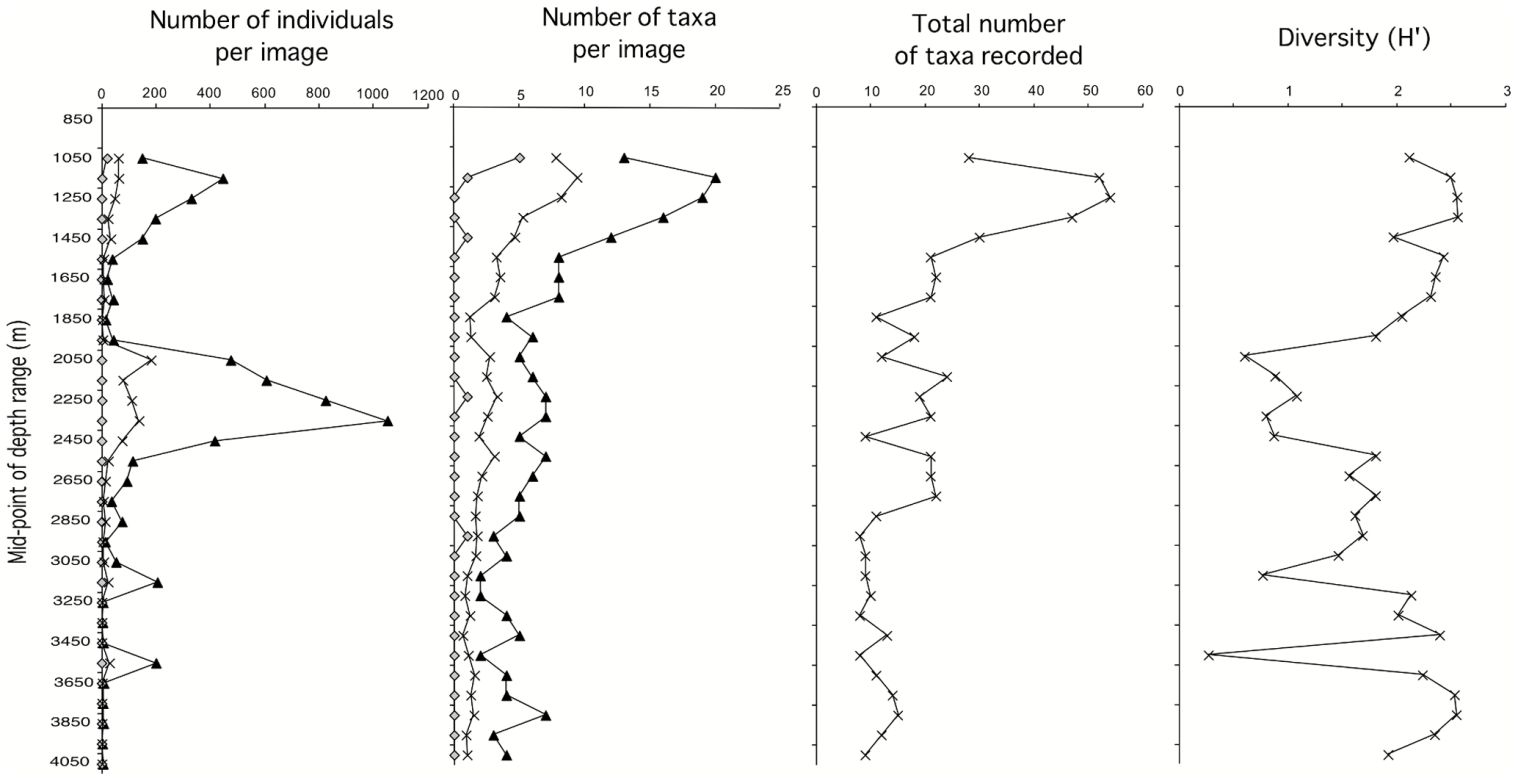
Number of individuals and number of identifiable taxa per image (minimum, maximum and mean values for both), total number of species recorded and diversity (H′) for images binned into 100 m depth strata.

Second, the abundance of megabenthos, as quantified by the number of individuals observed in the normalized images, correlates negatively with depth (r_s_ = 0.63, p<0.001), but was clearly not a uniform cline (Fig. 5). The highest counts were at intermediate depths, between 2000 and 2400 m, reaching a global maximum of an estimated 1052 organisms in a single image. There was a smaller peak in megabenthic abundance centered on 1100–1200 m. The shallow peak, coincident with the live *Solenosmilia* reef, reached a maximum of 446 organisms, i.e., 42.3% that of the deeper one. The deeper peak was due to the presence of large numbers of an unidentified hormathiid anemone, barnacles (*Tetrachaelasma tasmanicum*) and bamboo corals (Isididae). Over their respective depth zones (1000–1300 m vs. 2000–2400 m), the average number of organisms per image observed in the shallow peak was 51.6% that of the deeper one (65.7 vs. 127.18). Differences in abundance between the two peaks were not attributable to substratum type; sediment dominated only 1% (4 out of 405) of the scored images in the shallow peak as opposed to 9% (10 out of 107) in the deeper one.

The oblique and inconsistent image angle for the *Jason* light-bar camera makes calculation of absolute megabenthos density from those images difficult. However, peak densities could be calculated for the shallower (to 2995 m) depth range surveyed by *ABE*, whose down-ward looking camera photographed approximately 16 m^2^ in each image. The highest densities seen on *ABE* shallow surveys (820–1050 m), averaged over the whole image, were 33.2 organisms/m^2^ at 886 m (principally colonies of *E. rostrata*) and 17.1 organism/m^2^ at 1007 m (mainly *Pleurogorgia* spp.). The highest density recorded in a single towed camera image (area measure using stereo images) in this depth zone was 119.5 individuals/m^2^, at 1111 m, consisting mainly of *Stylaster* spp. and *Pleurogorgia* spp. By comparison, the highest average density values in a deep (>2000 m) *ABE* image was 42.3 organisms/m^2^, which consisted of 619 hormathiid anemones, 30 gorgonians and 27 barnacles, at 2056 m. Peak barnacle densities seen on the same dive (# 216) were 32.1 barnacles/m^2^ of rock substratum at 2171 m, and 29.7 barnacles/m^2^ rock substratum at 2184 m. Average overall density of live organisms for the latter image was 26.4 organisms/m^2^, consisting of 333 barnacles, 90 gorgonians and 2 hexactinellids. Most of the gorgonians in the image were small (<1 m high). Highest density of large gorgonians (*Isidella* and *Keratoisis* spp.), all estimated to be >1 m tall, was 2.25/m^2^ averaged over the whole image, at 2214 m.

Third, species richness, quantified as the mean number of taxa counted in each image declines significantly with depth (r_s_ = 0.84, p<0.0001), peaking coincident with the live *Solenosmilia* reef between 1100 and 1300 m (Fig. 5). The total number of species seen in each 100 m stratum correlates highly with the within-image richness (r_s_ = 0.77, p<0.001), declines with depth (r_s_ = 0.72, p<0.001) and is highest where the proportion of biogenic substrate is highest (r_s_ = 0.68, p<0.001) reflecting outlying high values for both parameters associated with the *Solenosmilia* reef. Over all depths, however, the total number of species seen also correlates with the number of images scored in each stratum (r_s_ = 0.84, p<0.001), even after excluding the shallow reef (for strata >1400 m, r_s_ = 0.83, p<0.001), suggesting that the trend in aggregated richness with 100 m depth strata could be at least partly an artifact of sampling effort; this limitation was overcome by aggregating multiple depth strata using multivariate analyses (see next section). Within-image diversity (H′) is also relatively uniform across depths (Fig. 5) (correlation between H′ and depth, r_s_ = 0.15, NS). The only depth range in which diversity was consistently low was between 2000 and 2600 m, coincident with the hormathiid/barnacle/bamboo coral peak in individual abundance. 95.1% of all organisms counted in this depth range were the hormathiid anemones.

**Figure 6:**
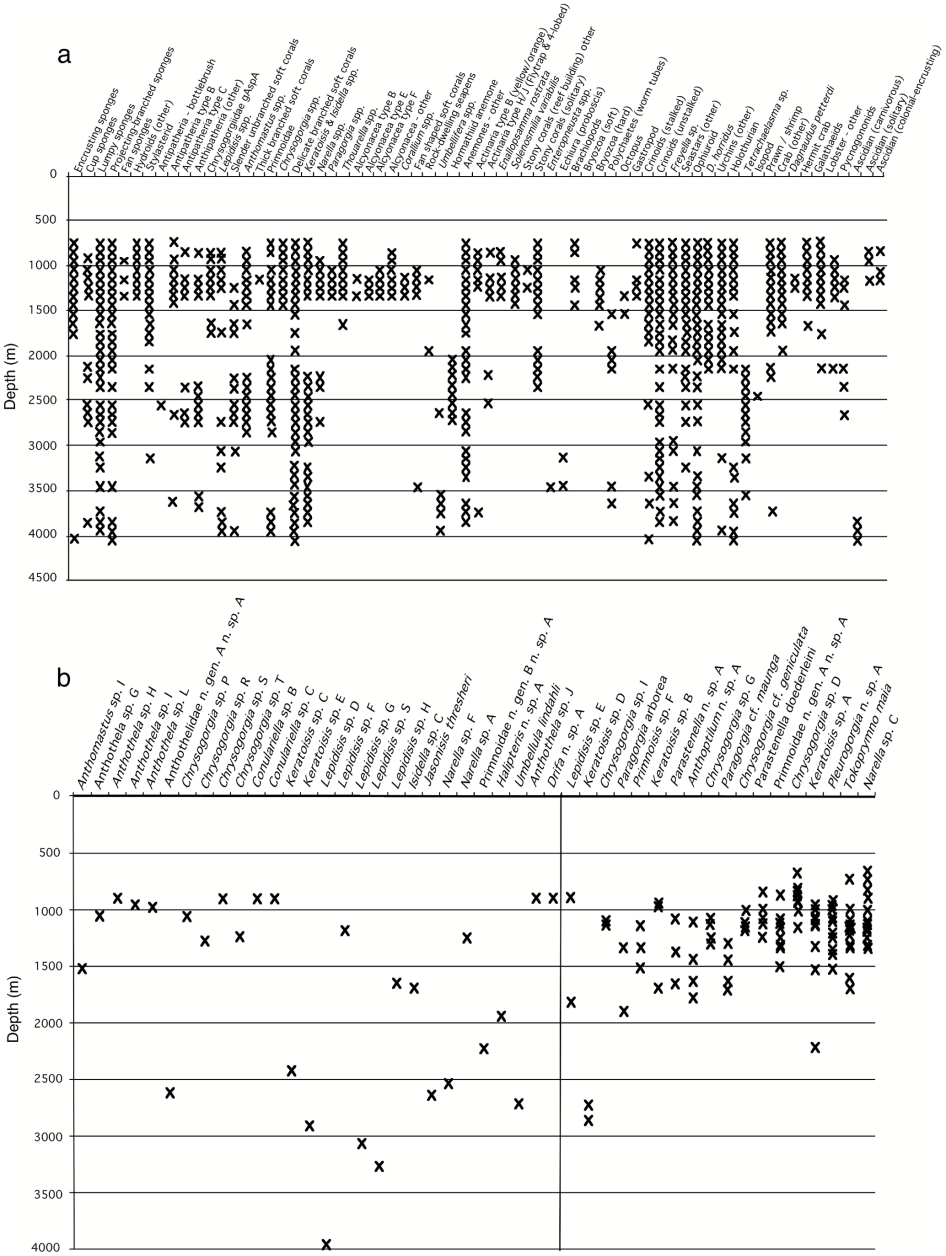
A. Depth distributions of identifiable megafaunal taxa in images taken the Tasmanian seamounts, binned by 100

Fourth, as implied above, the visually dominant taxa varied with depth. The colonial scleractinian corals (*S. variabilis*, *E. rostrata*) that dominated shallow depth ranges, and the hormathiids and barnacles that dominated between 2000 and 2500 m, all had distributional ranges ≤∼500 m. The vertical ranges for other taxa recorded in the images varied widely (Fig. 6a), but this could have been due, in part, to the limits of taxonomic resolution possible in photographic images. To test this, we examined the distribution records of the octocorals collected during the program in detail. Octocorals were specifically targeted for collection over a wide depth range during the *Jason* program and extensive shallow collections already existed; all were resolved to species-level in the laboratory. Their depth distributions are shown in Fig. 6b. Of 46 octocoral taxa collected, 28 (61%) were collected only once. This is despite half (14) having been collected shallower than 1500 m, in depths sampled relatively heavily by epibenthic sled. Among taxa collected more than once, 7 had a total depth range <500 m, and only 1 (*Keratoisis* sp. A) was collected over a depth range >1000 m.

Comparison of the depth-related trends in number of individuals seen per image, number of species and diversity, also show some strong non-linearities (Fig. 5). Thus, at the scale of 100 m depth strata, megabenthos density does not correlate with either the average number of species seen in each image or the summed species richness (r_s_ = 0.15 and r_s_ = 0.21, respectively, NS), reflecting an abundance distribution that is bimodal across depths and highest at intermediate depths, but species richness that is unimodally highest close to the seamount peaks and relatively uniform deeper. Abundance correlates negatively with H′ (r_s_ = 0.73, p<0.001), due to the dominance in images of a small number of conspicuous species in both the live *Solenosmilia* and the hormathiid/barnacle/bamboo coral strata (∼1000–1400 m depth and ∼2000–2400 m depth – Fig. 5). Aside from the latter stratum, diversity is relatively constant across the depths surveyed.

### Megabenthos assemblage structure

**Figure 7:**
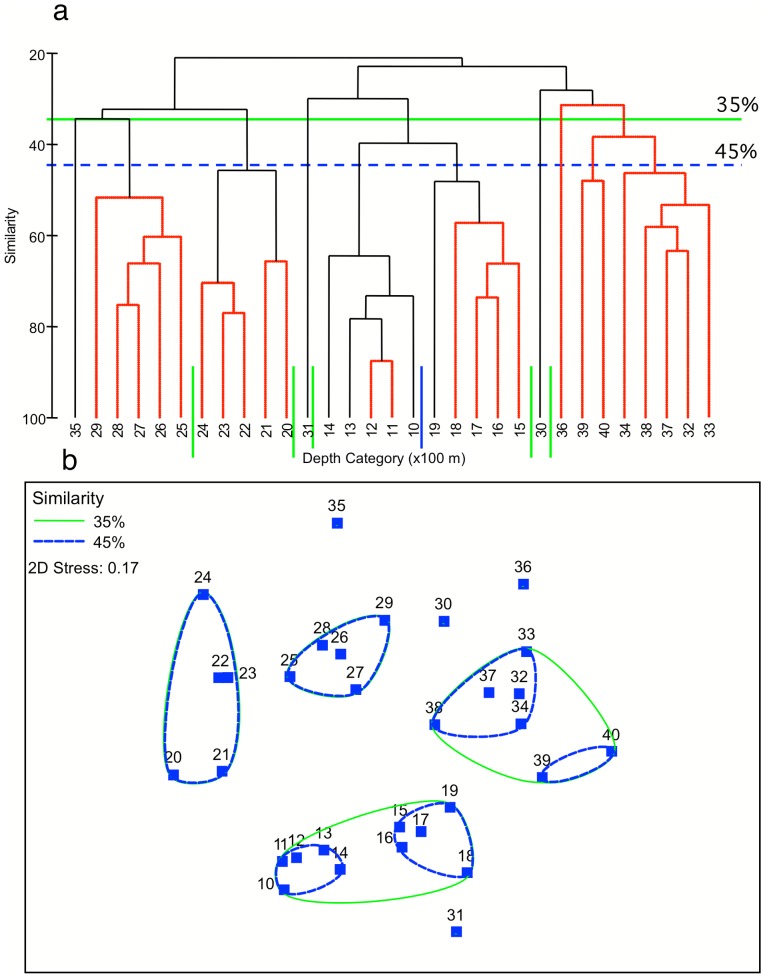
Results of cluster and MDS analyses of Tasmanian seamount biota binned by 100 m depth strata. Numbers indicate the upper depth of the strata X 100 (e.g., 6 = 600–700 m). The green and dashed blue horizontal lines in the cluster analysis and the outlines in the MDS indicate 35 and 45% levels of similarity, respectively, based on the Bray-Curtis similarity index of square-root transformed mean number of individuals in each identified taxon in images in each depth stratum. Vertical red lines in the cluster analysis indicate linkages that are interchangeable at the 95% confidence level. Clusters (see key) were identified at the 40% similarity level, except for the 3200–<4100 m group that showed no significant internal structure at 40% similarity.

**Figure 8:**
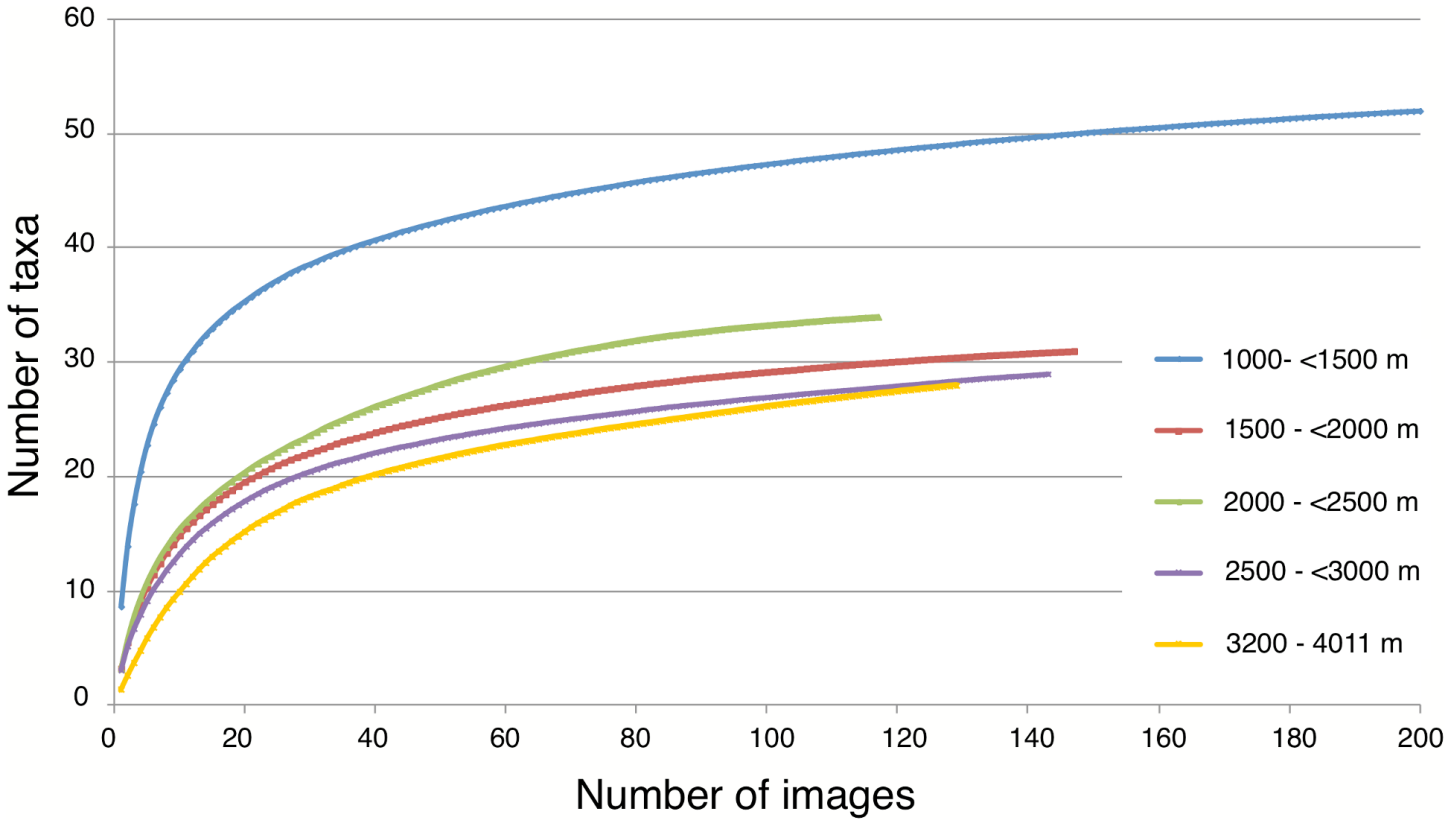
Average species accumulation curves for five depth zones, based on 999 random permutations of images in each zone.

Cluster analysis of multi-species distribution data (Fig. 7a) identified 6 assemblages at the 45% level of similarity that are grouped by depth and that form clearly identifiable clusters in the nMDS (Fig. 7b). The SIMPROF results show, however, that the variability within the deepest two of those 6 groups is not significantly less (p>0.05 as indicated with red lines) than between them, while 2 groups (1000–<1500 m and 2000–<2500 m) could be sub-divided further (Fig. 7a). Depth-stratified species accumulation curves, when aggregated into depth ranges coincident with the conspicuous bathymetric assemblages described above (Fig. 8), show that the five biotic zones were adequately sampled for comparison, as indicated by the flattening of the curves after approximately 100 images were sampled. Fig. 8 shows that the taxon richness does not gradually decrease with increasing depth of the biotic zones – the 1500–<2000 m zone is clearly less taxon diverse than the two zones either side of it. However, the curves also suggest that depth effects on aggregated species richness, below the *Solenosmilia*-dominated reef, are relatively small (Fig. 8).

The two outliers, deep strata that group closely with shallower strata 3100–<3200 and 3500–<3600, both had a relatively high maximum number of individuals per image and corresponding low H′ diversity (Fig. 5).

The six zones identified at 40% similarity (despite the non-significant difference between the deepest clusters), and their approximate depth ranges, are consistent with the qualitative observations made from all images and video footage collected using *ABE*, *Jason* and the towed camera, summarised below. The latter also indicate a seamount peak zone, that was not included in the semi-quantitative assays. The seven (*in toto*) depth-stratified zones are as follows:

(1) the *Enallopsammia* zone (seamount peaks to approximately <1000 m), characterised by high species richness, but a relatively low abundance of megabenthic organisms;

(2) the live *Solenosmilia* reef (1000–<1500 m) with high species richness and a relatively high abundance of organisms;

(3) a dead *Solenosmilia* and rock/rubble zone (1500–<2000 m), characterised by sparse live *Solenosmilia* at its shallow end and extensive areas of intact, but dead sub-fossil *S. variabilis* colonies and rock and rubble deeper, which appears relatively barren but has a scattered megabenthic and encrusting fauna;

(4) the anemone/barnacle/bamboo coral zone (2000–<2500 m) with a very high biomass, but low diversity, although the second highest cumulative taxon richness;

(5) a deep rock/rubble zone (2500–<3000 m) characterised by low individual abundance, moderate levels of species richness and diversity, fields of gorgonians on sloping rock surfaces and occasional multi-species patches of organisms;

(6) a deeper rock zone (3200–3900 m) of low individual abundance and species richness, widely scattered individuals and extensive barren areas; and below this

(7) a statistically similar, deep sand/rubble plain between 3900 and 4011 m, characterised by a scattering of, primarily, echinoderms.

### Descriptions of megabenthic assemblages in each depth zone

**Figure 9:**
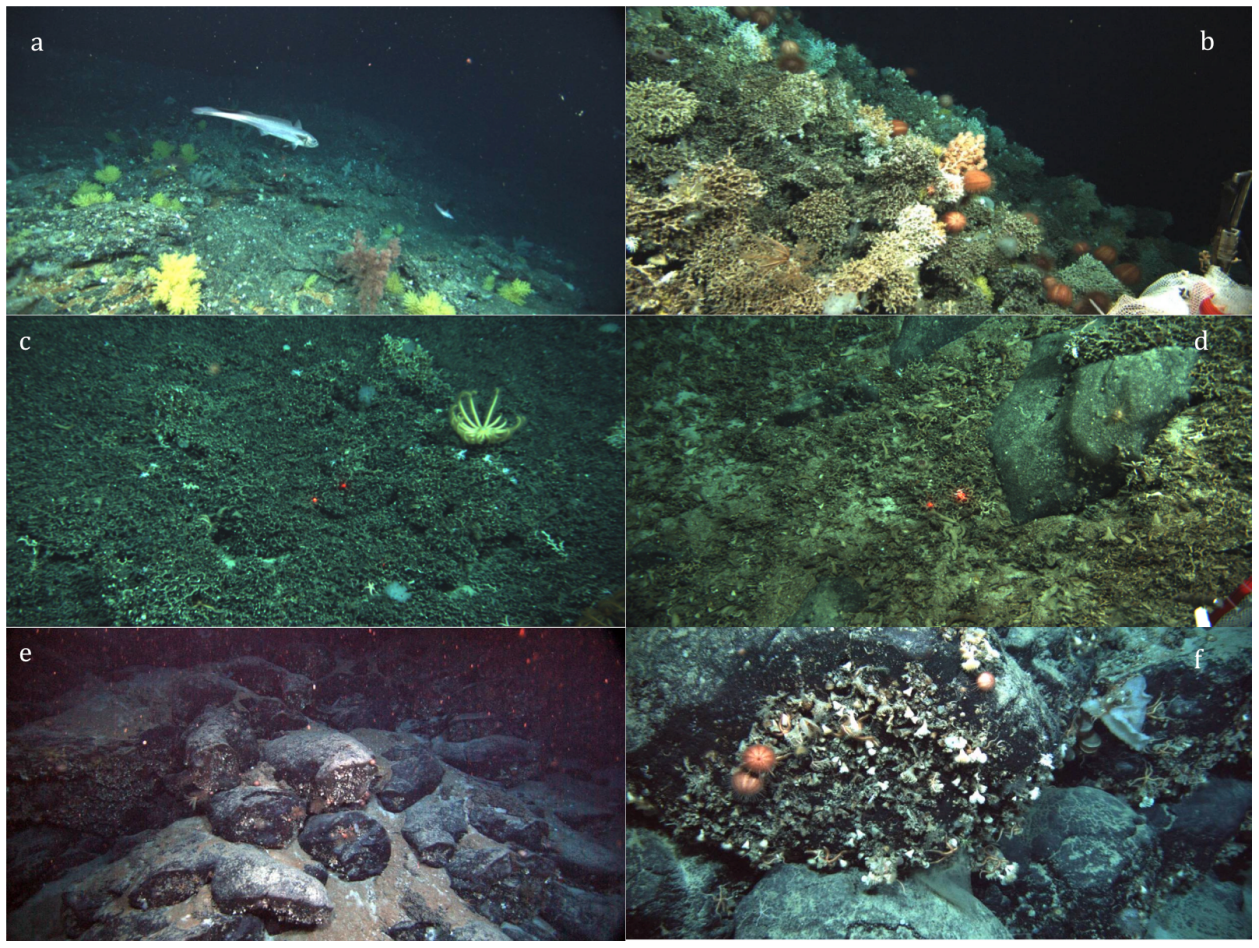
Representative images of the biotic zones on the Tasmanian seamounts. a. Blue grenadier (*Macroronus novaezelandiae*) swimming above the relatively barren rock substratum at 729 m on the peak of the Mongrel Seamount. A variety of gorgonian corals are visible on the bottom. *Jason* dive 386. b. The live *S. variabilis* reef, at about 1300 m on Seamount A1. *Jason* dive 382. c. Seascape of the dead *Solenosmilia* reef at 1480 m on Seamount A1. *Jason* dive 382. d. *Solenosmilia* rubble zone, at 1615 m on Seamount A1. *Jason* dive 383. e. Seamount A1, at 1530 m. The rock formation is pillow lava with fractured ends. f. Close-up of the side of a rock, showing a mix of epibenthic organisms attached to the rock, including solitary corals (*Desmosphyllum dianthus*), bivalves, and sponges, along with the motile ophiuroids and urchins. *Jason* dive 383. A large number of additional frame grabs taken during the *Jason* dives are available at http://4dgeo.whoi.edu/jason, under 2008 and tn228.

The shallowest portions of the seamounts surveyed, less than approximately 1000 m, have a sparse, but diverse biota, with extensive areas of “bare” rock and sandy substrata (Fig. 9a). This assemblage was observed by ROV on the Sisters and Mongrel Seamounts, and by towed camera surveys on 8 additional seamounts in and near the Huon CMR (Table 1). Most of these seamount peaks have been heavily trawled [16], [20]. The dominant reef-forming coral we observed in this zone was the low-growing *Enallopsammia rostrata*. Dead *E. rostrata* rubble also covered large areas to a depth of about 900 m to 1000 m, which formed a matrix on which a diverse biota was attached. Live *E. rostrata* was also observed on the peak of seamount Z27, at 1061 m, but was absent from deeper seamount peaks surveyed. The seamount peaks below this depth range that we observed, none of which had been trawled, were typically covered by very dense aggregations of the suspension-feeding urchin.

#### Dermechinus horridus

Between 1000 and 1300 m, the assemblage was dominated by live *Solenosmilia variabilis* reef, described previously by [16]. Our *Jason*-based observations of this assemblage were made primarily on seamounts A1, Mongrel, Z27 and K1, and supplement those reported in [20]. The reef consisted of a mixture of live and recently dead *S. variabilis* growing on top of a dense matrix of dead and sub-fossil *Solenosmilia* (Fig. 9b). The depth of the matrix was difficult to ascertain, but at one site where the reef had apparently been cut away by a dredge, we noted the matrix was at least 1.67 m thick [26].

A total of 301 species have been collected in this zone from the sled surveys [13], [17]. Common organisms present include brisingid sea stars, the urchin *Dermechinus horridus*, octocorals ranging from large Isidids and *Corallium* spp. to smaller *Chrysogorgia* spp. and diverse primnoids, various sponges, and non-stalked crinoids. Common interstitial species include galathaeid squat lobsters, shrimps, the recently described crab *Trichopeltarion janetae*, a variety of worms, the solitary corals *Desmophyllum dianthus* and *Caryophyllia* cf. *diomedeae*, and ophiuroids [see 27].

The deepest live *S. variabilis* observed in this program was a single small colony collected at 1458 m on seamount K1. Between roughly 1300 m and 1600 m, the reef structure was increasingly dominated by dead *S. variabilis*, most of which is blackened by ferro-manganese oxide deposits (Fig. 9c). Close observations on seamounts A1 and K1 indicated the dead coral largely maintained its structural integrity to about 1500 m and existed as extensive banks along the flanks of the seamounts (Fig. 9c). Our preliminary radiocarbon analyses of these dead specimens date them from 11–15 kyr BP. Below 1500 m, the substratum was a mixture of sand and *S. variabilis* fragments (Fig. 3d).

Megabenthic organisms were conspicuously sparse in the “dead reef” habitat and declined with increasing depth. Visually, the live biota was dominated by scattered small aggregations of the urchin *Dermechinus horridus*, the occasional brisingid sea star, and, along the lower fringe, xenophyophores. Within the reef matrix itself, we found small numbers of ophiuroids and occasional hydrocorals (stylasterids), sponges and solitary stony corals (*Desmophyllum dianthus* and *Caryophyllia* cf. *diomedeae*).

Between roughly 1500–1600 m, the dead *S. variabilis* reef became increasingly fragmented and intermixed with, on the one hand, open sand/rubble patches and, on the other, volcanic rock (Fig. 9e). The transition was marked by an increase in apparent species richness, with the rocks and sand rubble areas inhabited by diverse benthic organisms. The tops of the rocks and boulders were relatively barren, with most megabenthos attached to vertical or overhanging rock faces (Fig. 8f). Common organisms included live and dead solitary corals (*Desmophyllum dianthus*), bivalves (*Acesta saginata*), tube-forming serpulid polychaete worms, usually small hydrocorals (stylasterids), stalked crinoids, and glass sponges (Hexactinellida). Cnidarians other than stony corals were present, but usually in small numbers and as scattered individuals. These included a variety of *Anthomastus* spp., sometimes large anemones, and bamboo corals (Isididae). Colonies of the latter extended whip-like up to 2 m off the bottom. Ophiuroids and crinoids often colonized the larger branching organisms. Among motile biota, ophiuroids were common in this zone, along with the occasional octopus, sea star and a variety of urchins. Small barnacles were also commonly observed encrusting the skeletons of dead sponges. We also observed a number of large fish in this zone, including several very large rays, an undescribed bighead (Melamphaeidae) and macrourids. On more open, sandy areas in this depth range, we frequently encountered both benthic and pelagic holothurians, as well as a mix of urchins and ophiuroids.

**Figure 10:**
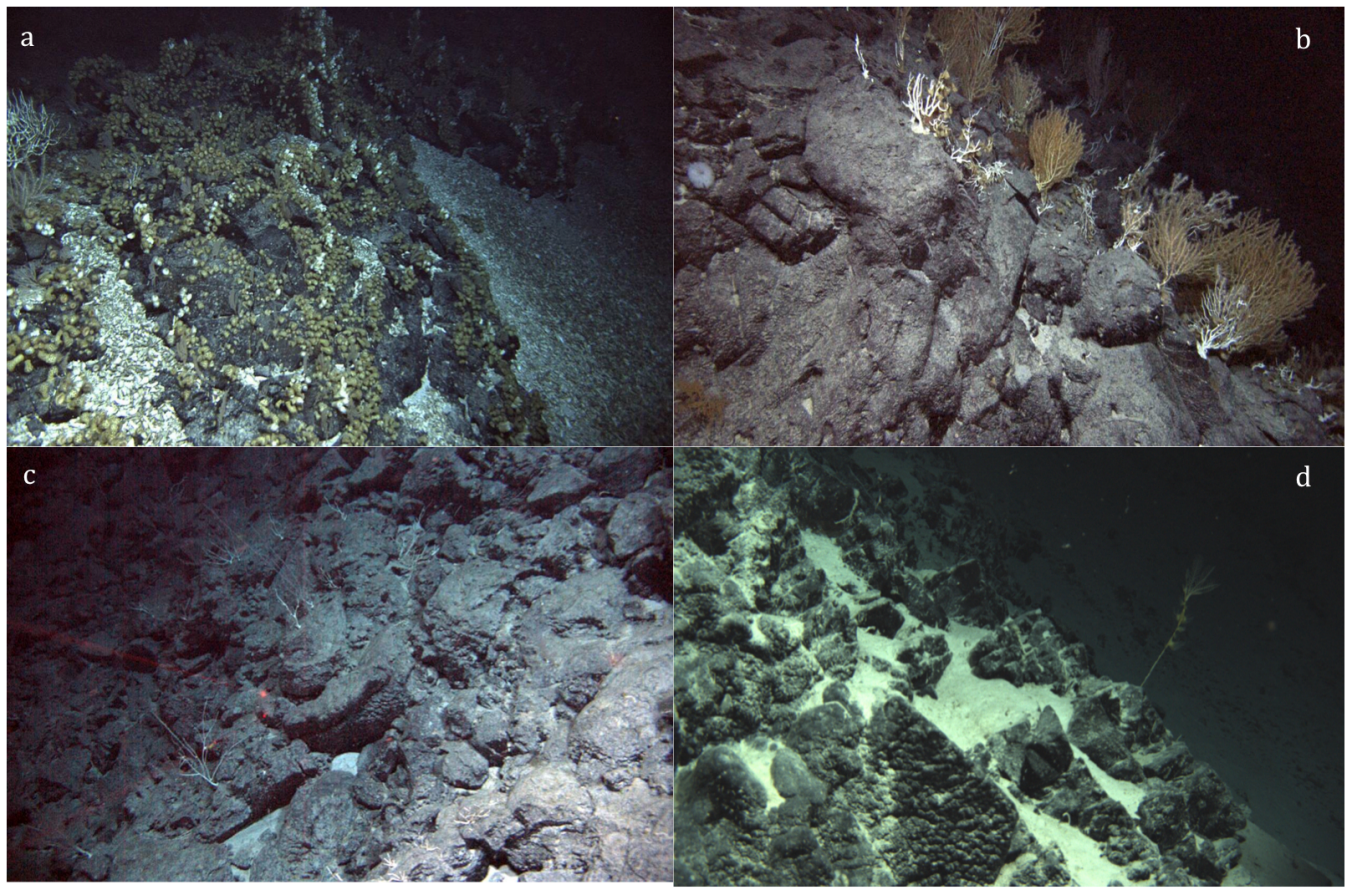
Representative images of the biotic zones on the Tasmanian seamounts. a. Hormathiid-dominated volcanic rock, at 2400 m. The rubble surrounding the rock is predominantly shell plates of deep-sea barnacles. Live barnacles are visible as white objects amongst the greenish-brown anemones, and to the left, a branching Isidid colony. For scale, the anemones are about 5 cm in diameter. b. Reef-scape at 2600 m, showing the dominance of live and recently dead (white skeletons) gorgonians, most apparently in the genus *Isidella*. Hormathiids (greenish brown) and barnacles (white) are visible on the rock, along with a glass sponge (Hexactinellid) to the left. Note the hormathiids attached to the up-right dead coral skeletons. c. Deep barren volcanic rock at 2820 m. The laser points are 10 cm apart. d. Interface between deep rock and sand plain at 3990 m. All photos were taken on *Jason* dive 392.

At depth of between 2000–2500 m, there was an abrupt increase in the abundance of benthic organisms, with the presence of three taxonomically unrelated groups: large, deep-sea barnacles, hormathiid anemones and large gorgonians, apparently in the isidid genera *Keratoisis* and *Isidella*. The barnacle has been tentatively identified as *Tetrachaelasma tasmanicum* [28]. Densities of organisms in this depth range can be extremely high, with hundreds of individuals often visible from *ABE* and *Jason* cameras [see 18]. Most available rock surfaces were largely covered by these organisms (Fig. 10a).

At depths approaching 3000 m, the number of conspicuous megabenthic organisms gradually decreased, with first the anemones, and then the barnacles largely disappearing. The area consisted of volcanic boulders and lava flow interspersed with ribbons of sand and rubble, on which there were typically few conspicuous organisms (Figs. 10b and c). Over most areas, the only prominent organisms were scattered gorgonian corals. Other than the gorgonians, the megabenthos in this depth range was very patchily distributed. On frequent occasions, while traversing the rock fields, small patches of a mixture of sponges, urchins, anemones, stalked crinoids, gorgonians and antipatharians were encountered. Although organisms were generally sparse, they were often large. Dominating one assemblage was a hexactinellid sponge, which stood about 2 m high and about 0.75 m across. These assemblages were generally confined to only a single boulder, and stood out strikingly from the surrounding largely barren expanses. The cause of this multi-species patchiness was not readily apparent.

At the base of the deep rock zone, observed only on our deepest dive (*Jason* 391), the rock fairly abruptly gave way to a sandy plain (Fig. 10d). The interface occurred between 3950 and about 3990 m. The rocks along the edge of the interface were inhabited by the occasional megabenthic organism, including an isidid (*Lepidisis* spp.) at 3945 m and a hexactinellid sponge at 3970 m. On the sloping sand plain beyond the rock/sand interface we observed ophiuroids, holothurians, pennatulaceans and a large, stalked ascidian (Octacnemidae).

**Figure 11:**
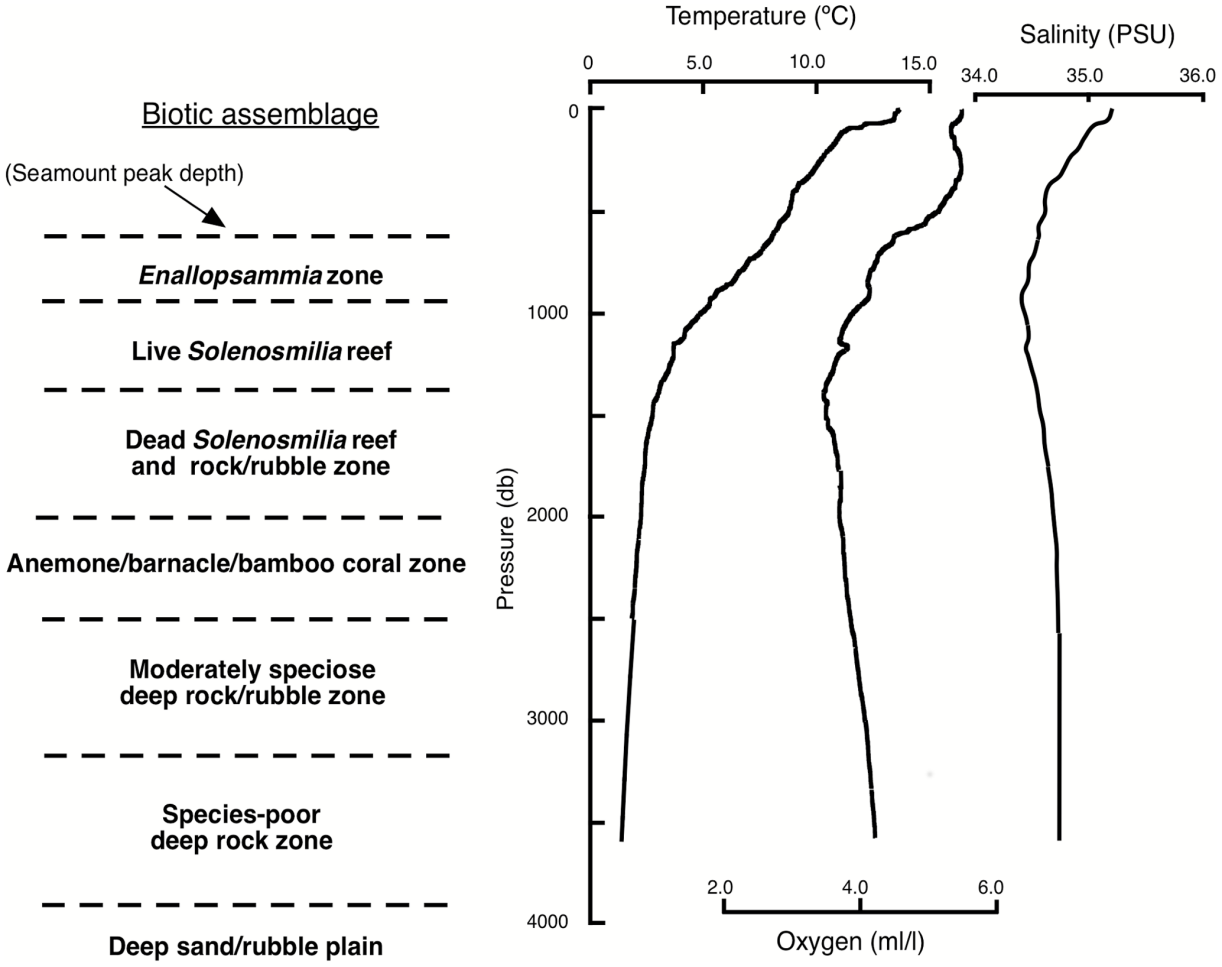
Diagrammatic representation of the seven principal biotic zones in the Marine Reserve Network, to a depth of 4 km. Temperature, salinity and oxygen profiles at 44° 50″ S, 145° 45″E on 10 Jan 2009, re-drawn from RV *Thomas G. Thompson* CTD cast 22802601.

There is a rough correspondence between vertical water mass distributions and the shallower zones observed (Fig. 11). The zones dominated by live bioherm-forming scleractinians (the *Enallopsammia* and *Solenosmilia* reefs) correspond to the depth of Antarctic Intermediate Water, centered at approximately 1000 m and characterised by a salinity minimum. The dead *Solenosmilia* reef and rock/rubble zone lies just below the oxygen minimum, at about 1400 m, which characterises the Upper Circumpolar Deep Water mass. There is no obvious temperature, salinity or oxygen feature associated with the high biomass barnacle/anemone/gorgonian zone, most of which would be in the Lower Circumpolar Deep Water mass.

## Discussion

### Depth zonation of megabenthic assemblages on the Tasmanian margin

Our semi-quantitative cluster and MDS analyses and qualitative observations collectively provide evidence of strong depth zonation in megabenthic assemblages on the southern Tasmanian continental margin between ∼700 and 4000 m depths. We identified seven such assemblages, which differ in dominant species (e.g., the *Enallopsammia* zone), live biomass cover (e.g., the live vs. dead *Solenosmilia* reef) or diversity (e.g., the anemone/barnacle/bamboo coral zone). Five of these zones were supported by results of the multivariate analyses. The shallowest zone was added based on observational data that could not be included in the semi-quantitative analysis, while the deepest zone was split into two based on a conspicuously different dominant biota. Transitions between these zones were visually relatively abrupt, typically spanning no more than 100–200 m. Proximally, the zonation is due in part to limited depth distributions for a few aspect-dominant species: *E. rostrata* appears to be a shallow seamount peak specialist, *S. variabilis* was abundant between 1100 and 1300 m, but rarely found alive below 1350 m, and both the hormathiid anemones and the abundant large barnacle (*T. tasmanicum*) were found only over a few hundred meter depth range centered on about 2300 m.

The shallow zones are represented by seamount tops with relatively sparse fauna and dominated by *E. rostrata* and *Pleurogorgia* spp., and appear to be widespread in our study area. Low diversity and biomass on shallow seamount peaks has been attributed to faunal removal by bottom trawling [17], [20], but our observations of fragile and intact specimens of *E. rostrata* and bamboo corals on seamount peaks suggest areas where overall diversity and abundance are naturally low. The slightly deeper, live *Solenosmilia* reef is also widespread, at around 1100 m depth off southern and NE Tasmania and New Zealand [21]. It is largely absent from the Cascade Plateau (pers. obs.) and the Lord Howe Rise [29], for reasons that are not immediately apparent. The few *S. variabilis* colonies we saw at the Cascade Plateau were small; several were dead. The deeper zone of extensive dead *Solenosmilia* also occurs across all the southern Tasmanian region sites we surveyed.

### Depth-related patterns in megafaunal assemblages of other deep rocky margins

Comparing depth zonation on the southern Tasmanian margin with that on other seamount-related habitats is difficult due to sparse information for most other regions [12], [30]. Sauta et al. [31], for example, describe communities on seamounts in the Andaman Sea, based on towed camera surveys from 373 to 2917 m, note large differences between seamounts, and attribute them primarily to substratum characteristics. Although the five transects in this study span more than a 2.5 km depth range, limited replication constrains any potential bathymetric analysis. Waller et al. [32] similarly describe diverse megabenthic communities on three seamounts in the Southern Ocean (Drake Passage), based on towed camera images to 1900 m. They suggest these differences relate to turbidity, temperature and substrata. Although they did not explicitly factor bathymetry into their analyses, they did note that sites on two seamounts that clustered relatively closely together spanned similar depth ranges, acknowledging the likely importance of depth as a direct or indirect structuring factor.

Preliminary work on northwest Atlantic seamounts [33] suggests depth zonation for seamount communities on the New England Seamount Chain and the Corner Rise Seamounts, with multidimensional scaling and cluster analyses detecting bathymetric breaks in community composition at 1300 m, 2300 m, and 2600 m [17]. More information is available for a few eastern Pacific seamounts [13], [15], [34]. Patterns of megabenthos distribution on several seamounts in similar depths to those we surveyed were broadly consistent with our observations, e.g., relatively shallow dominance of scleractinians with wider and deep ranging antipatharians and gorgonians [34]. Three depth-related patterns in the structure of megabenthos on Davidson Seamount in1246–3656 m depths were reported by McClain et al. [13]. These authors found no significant trends in density, species richness or diversity across depth, but increasingly divergent species composition with increased depth, with biotic zones stratified by depth (three zones) or substrate characteristics (one zone).

Some, but not all of these trends observed in the eastern Pacific are evident on the Tasmanian seamounts. The correlation across depth strata between the relative abundance of non-sediment substrata and the proportion of those images with identifiable megafauna, for example, evidences an effect of substrate characteristics on the Tasmanian assemblage structure, but was not something we specifically factored into our analyses because of the overall predominance of rock, rubble and biogenic substrates across depths. Similarly, the relatively narrow depth ranges of both the aspect dominant taxa and most species of octocorals implies increasingly divergent taxonomic overlap with increasing depth differences that is consistent with observations by McClain et al. [13]. Our observations differ in several key aspects, however. We note seven conspicuous biotic depth zones, as opposed to three, and non-linear trends with depth in abundance, richness and diversity that reflect specific characteristics of particular assemblages, e.g., a rich biota associated with the *Solenosmilia* reef and low diversity but very high abundances in the hormathiid/barnacle/bamboo coral zone. Despite the non-linearities, however, our data support broad suggestions that species richness and diversity, at least, do not decline markedly with depth in seamount communities.

### Constraints on species' depth distributions

Identifying the factor(s) that constrain the distribution of a deep-sea species requires either logistically difficult experimental studies or detailed comparative analyses of distributions across habitats that differ in environmental and depth ranges [37]. The depth distributions of cold-water corals in general have been suggested to be primarily a function of water temperature [30], interacting with features such as the availability of suitable rocky substrata, currents favorable to concentrating planktonic or particulate food items, and the depth of the deep scattering layer [30], [32], [39]. The lower depth limits for bioherm-forming scleractinians and octocorals have also been attributed to the depth of the aragonite and calcite saturation horizons, respectively [37], [40], [41]. However, direct evidence that tests any of these hypotheses is lacking. We noted that the maximum depth at which we saw live specimens of the solitary scleractinian *Desmophyllum dianthus*, identified from imagery and samples, was 2384 m at the Cascade Plateau (Tasman Sea) and 2395 m off SW Tasmania, a similarity which suggests the species may be constrained directly by pressure [3] or an environmental variable closely correlated with pressure, e.g., aragonite saturation state. Limited taxonomic resolution for what is largely an undescribed biota and the selectivity of the deeper sampling makes it difficult to determine the extent to which other, less easily identified taxa exhibit similarly consistent depth ranges. Our analysis of the collected octocoral fauna suggests a maximum depth range for any single species (*Keratoisis* sp. A) of about 1000 m, typical depth ranges for species collected ≥2 times of 300 – 600 m, and a suite of species constrained to depths less than about 1600 m (see Fig. 5b). This latter depth could reflect an environmental boundary, as it coincides with the transition between the dead *Solenosmilia* reef and a deeper rock/rubble zone. However, it also coincides with the transition between dredge-based sampling and selective sampling using *Jason*, and hence could be partly a sampling artifact. Relatively broad distributions are also suggested for Tasmanian seamount ophiuroids, another relatively well studied group [27]. The ophiuroid samples span a smaller depth range than ours (to a maximum of only 2003 m) and are trawl based and hence less precise, but of the twelve taxa identified, only two were shallow specialists (maximum depth ≤1450 m) and hence possibly associated with the *Enallopsammia* and *Solenosmilia* zones. No ophiuroid species were found exclusively below these zones, however, the average depth range for a single species was over 700 m, and four species occupied virtually the entire depth range sampled.

On a more gross taxonomic scale, trends with depth are mixed. Bioherm-forming scleractinians disappear at about 1400 m, solitary ones at 2400 m, and some conspicuous octocoral genera (e.g., *Corallium*, *Chrysogorgia* and *Paragorgia*), as well as brachiopods and bryozoans, by 2000 m, whereas large barnacles and hormathiid anemones only appear below 2000 m and then disappear by 3000 m. In contrast, many taxa span nearly the entire depth range we surveyed, including antipatherians, crinoids, ophiuroids and holothurians. Specimens of the octocoral genus *Lepidisis* have been collected in the Tasmanian region as shallow as 635 m. We saw it abundantly on the seamount peaks. However, we also collected one at 3945 m. Similarly, we photographed a specimen of the common seamount peak octocoral genus *Narella* at 3893 m. Our data suggest that major taxonomic shifts define and differentiate depth-related megabenthic assemblages, but that shifts overlay a commonality to much of the fauna associated with seamount and rocky substrate across the depth range examined.

### Mechanisms supporting the deep water biomass peaks

A major difference between our observations and those for deepwater communities in general [35], [36] is the non-linearity with depth in megafaunal abundance and apparent biomass. On the Tasmanian seamounts, biomass densities peak twice – once at the intermediate depths dominated by the colonial scleractinians and again nearly a kilometer deeper in the zone dominated by hormathiid anemones, barnacles and gorgonians. The abundance of live organisms in these two peaks is roughly similar, but wet weight biomass in the hormathiid-dominated zone is more than 10 times higher than for the *Solenosmilia* reef [18].

The proximate causes of the bathymetric structure of Tasmanian seamount assemblages are not immediately obvious. The presence of a high biomass and diverse biota near the seamount peaks is consistent with a carbon source for the assemblage that is based on surface-derived sinking particulates, as widely suggested for deep sea biota in general [4]. Carbon isotope analyses generally support this hypothesis locally, with indications of surface-sourced bomb radiocarbon in tissue samples to a depth of at least 3945 m, and most ∂^14^C values that are similar to and parallel the trend in particulate organic carbon (POC) ∂^14^C towards lower values in deeper water [18]. However, how this dependency on surface production results in the highest biomass densities on Tasmanian seamounts occurring at 2000 – 2600 m, well below the seamount peaks, is unclear. High biomass benthic assemblages in very deep water elsewhere are associated with alternative or concentrated but ephemeral carbon sources, e.g., hydrothermal vents [8], [38] and whale carcasses [35], neither of which appears to be relevant on the Tasmanian seamounts. Whether there are oceanographic factors that focus surface-derived particulates at 2000–2600 m is not known and the data to test for such water column structuring sparse. Druffel & Bauer [42] report POC concentrations to a depth of 5441 m in the open Southern Ocean south and east of the Tasmanian seamount region, but find no indication of a peak at or near 2000 m (E. Druffel pers. comm.). However, Honjo et al. [43] report very high failure rates, due to clogging, of sediment traps moored at 1976–2966 m, but not at about 1000 m or just above the bottom (>3000 m), at sites in the Polar Frontal Zone, also south and east of the Tasmanian region. This clogging could indicate a deep stratum of high particle densities. Lateral transport of subducted polar production equatorwards is not implausible given general Southern Ocean circulation patterns [44]. Alternatively, the gap between the shallow and deeper biomass peaks on the seamounts could reflect a continuously high availability of particulate food, but depressed biotic densities between 1500 and 2000 m due to otherwise adverse environmental conditions. In that regard, we note that this low density zone is just below the depth of the water column oxygen minimum. Low oxygen levels have been reported to adversely affect the growth and survival of a shallow cold-water coral in the North Atlantic [45] and body sizes in some deep-sea taxa [46], [47]. Very low oxygen levels are known to severely constrain the distributions and diversity of a range of deep-sea taxa [10], [39]. The oxygen minimum zone also coincides with sparse populations of the solitary scleractinian *Desmophyllum dianthus* between 1500–2000 m on Tasmanian seamounts [48].

Such hypotheses remain speculative in the absence of complementary experimental or oceanographic studies or detailed distributional data for other sites against which the Tasmanian data can be compared. Our analyses, nonetheless, demonstrate that bathymetric structuring of seamount communities can be more complex than a simple monotonic decline in assemblage parameters with increasing depth. The presence of these deep assemblages highlights the potential for unforeseen negative consequences of rapidly developing deep-sea exploitation in unexplored national and international waters [5], [19]. Our data suggest that for seamount communities, at least, an assumption that biota at depths in excess of 1–2 km is sparse is not warranted.
